# Cannabinoid Receptor 2 Modulates Maturation of Dendritic Cells and Their Capacity to Induce Hapten-Induced Contact Hypersensitivity

**DOI:** 10.3390/ijms21020475

**Published:** 2020-01-11

**Authors:** Evelyn Gaffal, Andrea M. Kemter, Stefanie Scheu, Rafael Leite Dantas, Jens Vogt, Bernhard Baune, Thomas Tüting, Andreas Zimmer, Judith Alferink

**Affiliations:** 1 Department of Dermatology, University Hospital Magdeburg, 39104 Magdeburg, Germany; evelyn.gaffal@med.ovgu.de (E.G.); thomas.tueting@med.ovgu.de (T.T.); 2 Institute of Molecular Psychiatry, University of Bonn, 53127 Bonn, Germany; akemter@uchicago.edu (A.M.K.); a.zimmer@uni-bonn.de (A.Z.); 3 Department of Pathology, The University of Chicago, Chicago, IL 60637, USA; 4 Institute of Medical Microbiology and Hospital Hygiene, University of Düsseldorf, 40225 Düsseldorf, Germany; stefanie.scheu@uni-duesseldorf.de; 5 Department of Psychiatry, University of Münster, 48149 Münster, Germany; rafael.leitedantas@ukmuenster.de (R.L.D.); Vogt_Jens88@web.de (J.V.); bernhard.baune@ukmuenster.de (B.B.); 6 Department of Psychiatry, The University of Melbourne, Melbourne 3010, Australia; 7 The Florey Institute of Neuroscience and Mental Health, The University of Melbourne, Melbourne 3010, Australia; 8 Cells in Motion Interfaculty Centre, 48149 Muenster, Germany

**Keywords:** allergic contact dermatitis, dendritic cells, skin inflammation, CB1, CB2, cannabinoid receptors, hapten 2,4-dinitro-1-fluorobenzene, MHC, migration, CCL19, CXCL12

## Abstract

Contact hypersensitivity (CHS) is an established animal model for allergic contact dermatitis. Dendritic cells (DCs) play an important role in the sensitization phase of CHS by initiating T cell responses to topically applied haptens. The cannabinoid receptors 1 (CB1) and 2 (CB2) modulate DC functions and inflammatory skin responses, but their influence on the capacity of haptenized DCs to induce CHS is still unknown. We found lower CHS responses to 2,4-dinitro-1-fluorobenzene (DNFB) in wild type (WT) mice after adoptive transfer of haptenized *Cnr2*^−/−^ and *Cnr1^−/−^/Cnr2^−/−^* bone marrow (BM) DCs as compared to transfer of WT DCs. In contrast, induction of CHS was not affected in WT recipients after transfer of *Cnr1*^−/−^ DCs. In vitro stimulated *Cnr2^−/−^* DCs showed lower CCR7 and CXCR4 expression when compared to WT cells, while in vitro migration towards the chemokine ligands was not affected by CB2. Upregulation of MHC class II and co-stimulatory molecules was also reduced in *Cnr2^−/−^* DCs. This study demonstrates that CB2 modulates the maturation phenotype of DCs but not their chemotactic capacities *in vitro*. These findings and the fact that CHS responses mediated by *Cnr*2*^−/−^* DCs are reduced suggest that CB2 is a promising target for the treatment of inflammatory skin conditions.

## 1. Introduction

Allergic contact dermatitis (ACD) is an inflammatory skin reaction to subthreshold exposures to low-molecular-weight compounds called haptens [1]. Skin manifestations such as pruritic skin lesions at sites of allergen exposure often manifest chronically, and therefore they represent a rising global burden on the healthcare system [2,3].

A well-established experimental murine model of ACD is 2,4-dinitro-1-fluorobenzene (DNFB)-induced cutaneous contact hypersensitivity (CHS). The inflammatory response in CHS develops in two distinct phases [4,5]. In the sensitization phase, activation of epidermal Langerhans cells and dermal dendritic cells (DCs) is promoted by haptenized proteins [4]. Maturation-induced migration of antigen-presenting cells into the regional lymph nodes for initiation of hapten-specific T cell responses along chemokine gradients involves sequential upregulation of chemokine receptors [6]. On the other hand, recent findings have suggested that DCs may also be required for the maintenance of immune tolerance in CHS [7]. In the elicitation response, re-challenge with DNFB provokes a regulated skin immune response associated with effector T cell recruitment, which peaks 24 h post challenge [8].

The endocannabinoid system includes the cannabinoid receptors, their endogenous ligands (endocannabinoids), and cannabinoid synthesizing and metabolizing enzymes [9]. Cannabinoid receptor 1 (CB1), to which the psychotropic effects of 9-tetrahydrocannabinol (9-THC) have been attributed, is expressed mainly in the central nervous system [10,11,12,13,14]. In contrast, cannabinoid receptor 2 (CB2) is expressed primarily by immune cells and has been found to play a key immunomodulatory role in various physiological and pathophysiological conditions [10,11,12,15,16,17,18,19]. Interestingly, CB1 and CB2 are expressed in keratinocytes [20,21] and are involved in their proliferation and differentiation [22]. Moreover, we and others have demonstrated that CB1 and CB2 and their endogenous ligands are involved in skin allergies. Genetic deletion of both CB1 and CB2 enhances contact allergic inflammation, with non-redundant roles for each cannabinoid receptor in the allergic response to DNFB [21]. On the other hand, CB2-specific antagonists/inverse agonists have been reported to alleviate chronic inflammation in mice, suggesting that there may be complex CB2-dependent regulation of inflammatory skin immune responses [23,24].

CB2 signaling can further affect the basic functions of DCs, which represent key cellular players in allergic skin responses. A prior study demonstrated that treatment with a CB2 agonist inhibited DC migration towards CCL19 and the expression of matrix metalloproteinase 9 [25]. We showed previously that lipopolysaccharide (LPS)-stimulated bone marrow (BM) derived-dendritic cells from *Cnr2^−/−^* mice secrete higher levels of several cytokines, including tumor necrosis factor (TNF), interleukin (IL)-6 and IL-12, as well as transforming growth factor-β (TGF-β) [26]. However, the specific role of CB2 in modulating DC-dependent sensitization for CHS has not yet been studied. Additionally, while the protective effects of CB1 in CHS have been attributed to its presence on keratinocytes [27,28], a potential role of CB1 in DCs has not been assessed up to now.

In this study, we examined the specific influence of CB1 and CB2 on the capacity of DCs to induce CHS and the specific role of CB2 in DC maturation and migration.

## 2. Results

### 2.1. Reduced Contact Hypersensitivity upon Adoptive Transfer of Haptenized Cnr2^−/−^ Dendritic Cells

**Figure 1:**
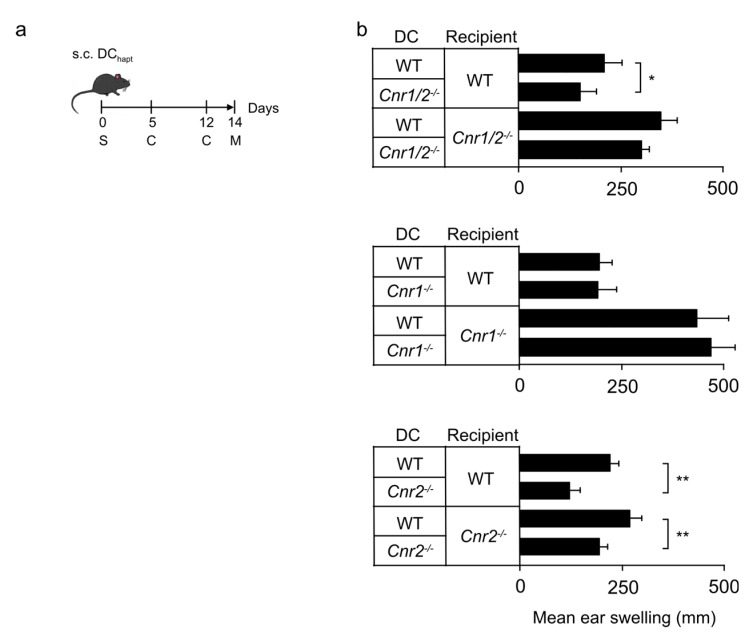
Reduced hapten-induced contact hypersensitivity upon transfer of cannabinoid receptor 2 (CB2)-deficient dendritic cells (DCs) into recipient mice. (**a**) Mice were sensitized (S) by subcutaneous (s.c.) injection of haptenized DCs and challenged (C) with 2,4-dinitro-1-fluorobenzene (DNFB). Ear swelling (M) was measured 48 h after the second challenge. (**b**) Mean ear swelling 48 h after the second challenge with DNFB (± SEM, *n* = 5 mice/group) in wild type (WT), *Cnr1/2*^−/−^, *Cnr1*^−/−^ and *Cnr2*^−/−^ mice sensitized with WT DCs, *Cnr1*^−/−^*/Cnr2*^−/−^ DCs (top), *Cnr1*^−/−^ DCs (middle) and *Cnr2*^−/−^ DCs (bottom). Experiments were repeated two times with similar results. * *p* < 0.05, ** *p* < 0.01.

We utilized an in vivo adoptive transfer model to study the effect of CB1 and/or CB2 deficiency on the capacity of haptenized DCs to sensitize naïve mice for contact hypersensitivity (CHS). For this, we injected DNFB-haptenized DCs generated from the bone marrow (BM) of *Cnr1^−/−^/Cnr2^−/−^*, *Cnr1^−/−^*, *Cnr2^−/−^* or wild type (WT) mice into naïve WT recipients. Animals were subsequently challenged twice with DNFB and ear swelling was measured after 48 h (Figure 1a). DNFB-challenged WT mice showed allergic ear swelling after sensitization with haptenized WT DCs (Figure 1b). However, the CHS response was significantly reduced in mice sensitized with haptenized *Cnr1^−/−^/Cnr2^−/−^* DCs as compared to recipients injected with WT DCs. A reduced ear swelling reaction to DNFB was also observed in WT animals after adoptive transfer of *Cnr2^−/−^* DCs, whereas induction of CHS was not affected in *Cnr1^−/−^* DC-treated WT recipients.

To investigate whether CB1 and CB2 also modulated the response of the host, haptenized DCs were injected into *Cnr1^−/−^/Cnr2^−/−^*, *Cnr1^−/−^*, and *Cnr2^−/−^* recipient mice as well as into WT controls. Again, recipient mice were subsequently challenged twice with DNFB (Figure 1a). Adoptive transfer of WT cells into *Cnr1^−/−^/Cnr2^−/−^* or *Cnr1^−/−^* mice, but not *Cnr2^−/−^* mice, induced exacerbated allergic responses when compared to WT recipients (Figure 1b). These findings indicate that CB1 affects the host immune response during CHS, while CB2 expression on DCs in the sensitization phase alters their ability to initiate CHS responses.

Thus, CB1 signaling in DCs is dispensable during naive T cell priming but is critical in host cells. We therefore focused our subsequent analyses on the specific role of CB2 in DC maturation and migration.

### 2.2. Reduced Expression of Chemokine Receptors on Cnr2^−/−^ and WT BM-DCs

**Figure 2:**
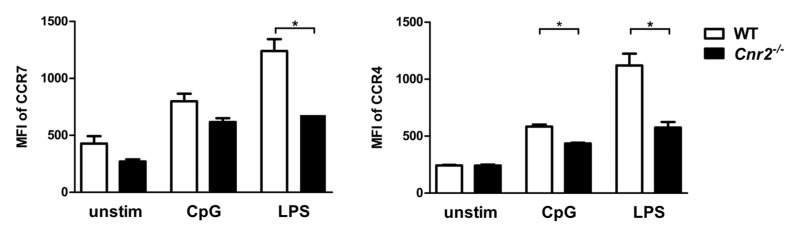
Reduced expression of the chemokine receptors CCR7 and CXCR4 on activated CB2-deficient BM-DCs. BM-DCs from WT (white bars) or *Cnr2*^−/−^ (black bars) mice were cultured for 16 h in the absence (unstim) or presence of 1 nmol/mL CpG or 100 ng/mL lipopolysaccharide (LPS). Surface expression of CCR7 and CXCR4 was analyzed by flow cytometry. Data show median fluorescence intensity (MFI) ± SEM (*n* = 4–5 animals/group). * *p* < 0.05.

It is well established that the chemokine receptors CCR7 and CXCR4 and their cognate ligands CCL19/CCL21 and CXCL12, respectively, are regulators of skin DC migration under inflammatory conditions [29,30]. We therefore assessed expression of CCR7 and CXCR4 on BM-DCs of both genotypes by flow cytometry. Levels of both chemokine receptors did not differ between WT and *Cnr2^−/−^* DCs (Figure 2). However, upon Toll-like receptor 4 (TLR4) stimulation with LPS, upregulation of CCR7 expression was significantly lower on *Cnr2^−/−^* DCs when compared to WT cells. Furthermore, *Cnr2^−/−^* DCs exhibited lower CXCR4 expression levels as compared to WT cells after TLR4 or TLR9 ligation with LPS or CpG oligonucleotide 1668 (CpG), respectively (Figure 2).

**Figure 3:**
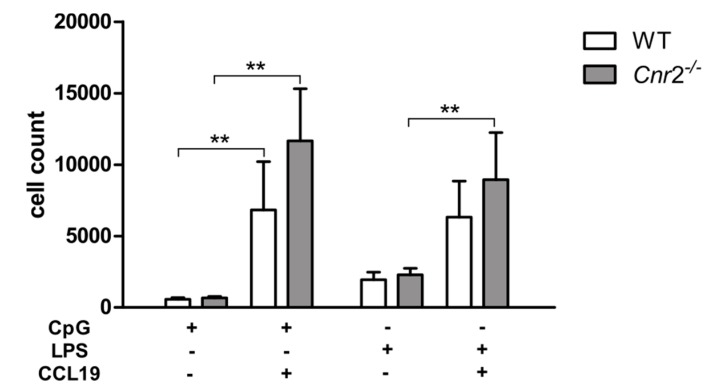
In vitro migration of activated BM-DCs towards CCL19 is not affected by CB2 deficiency. Migratory behavior of BM-DCs from WT (*n* = 5) and *Cnr2*^−/−^ (*n* = 5) mice in transwell migration assays in response to 200 ng/mL CCL19 or medium only. CpG or LPS activated WT or *Cnr2*^−/−^ BM-DCs were seeded in the upper transwell chambers. After 4 h cells were collected in the bottom well and cell counts determined. Data show mean values ± SD (*n* = 4–5 animals/group). ** *p* < 0.01.

To determine whether CB2 affects migration of DCs, we next examined the chemotactic capacity of WT and *Cnr2*^−/−^ DCs using in vitro transwell assays. For this, LPS or CpG stimulated BM-DCs were loaded into the upper well with or without addition of CCL19 in the bottom well. While migration of DCs was greatly increased in the presence of CCL19, WT and *Cnr2*^−/−^ BM-DCs migrated toward CCL19 in equivalent numbers (Figure 3). These data demonstrate that CB2 deficiency does not alter the chemotactic behavior of DCs in response to CCL19.

### 2.3. Reduced Expression of MHC Class II (MHC II) and Co-Stimulatory Molecules by Cnr2^−/−^ BM-DCs upon TLR Stimulation

**Figure 4:**
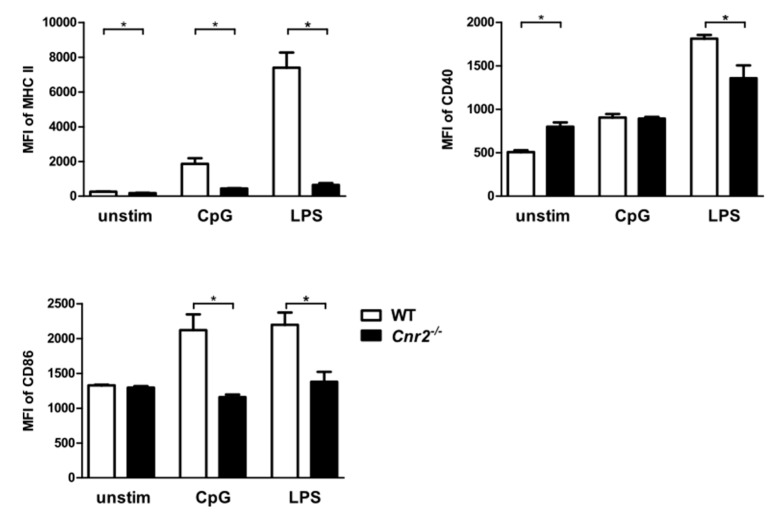
Reduced surface expression of MHC II and co-stimulatory molecules by *Cnr2*^−/−^ BM-DCs after Toll-like receptor (TLR) stimulation. Surface expression of MHC II, CD40, and CD86 by WT (white bars) or *Cnr2*^−/−^ (black bars) BM-DCs cultured for 16 h in the absence (unstim) or presence of 1 nmol/mL CpG or 100 ng/mL LPS. Data show median fluorescence intensity (MFI) ± SEM (*n* = 4–5 animals/group). * *p* < 0.05.

A prerequisite for CHS is the maturation of DCs during their migration to the draining LNs following exposure to antigen. We therefore examined the influence of CB2 on the maturation of BM-DCs from *Cnr2^−/−^* and WT mice by analyzing surface expression of MHC II and the co-stimulatory molecules CD40 and CD86 upon stimulation with LPS or CpG. We found reduced expression of MHC II on BM-DCs isolated from *Cnr2^−/−^* mice in comparison to WT BM-DCs in the naïve state and upon stimulation. Notably, higher expression levels of CD40 were observed in naïve *Cnr2^−/−^* BM-DCs when compared to WT BM-DCs. However, stimulation with LPS induced lower upregulation of CD40 on *Cnr2^−/−^* BM-DCs when compared to WT cells (Figure 4). In addition, CD86 expression was markedly enhanced in WT BM-DCs upon stimulation with CpG, while it was not affected in *Cnr2^−/−^* BM-DCs. Thus, CB2 affects upregulation of MHC II and co-stimulatory molecules upon maturation of DCs, a process required for naïve T cell stimulation.

**Figure 5:**
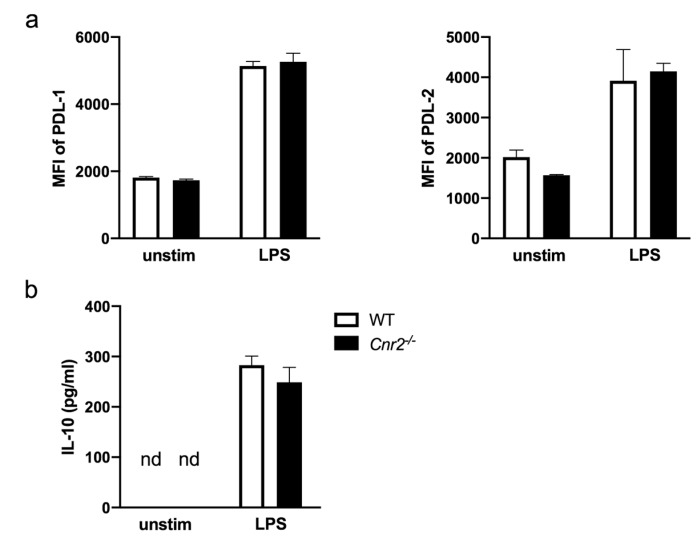
Equivalent surface expression of programmed death-ligand 1 (PD-L1), PD-L2 and IL-10 production by *Cnr2*^−/−^ and WT BM-DCs after TLR4 stimulation. (**a**) Surface expression of PD-L1 and PD-L2 by WT (white bars) or *Cnr2*^−/−^ (black bars) BM-DCs cultured for 16 h in the absence (unstim) or presence of 100 ng/mL LPS. (**b**) Production of IL-10 by WT (white bars) or *Cnr2*^−/−^ (black bars) BM-DCs cultured for 16 h in the absence (unstim) or presence of 100 ng/mL LPS. Data show median fluorescence intensity (MFI) ± SEM (*n* = 3-4 animals/group).

Finally, we investigated possible CB2-mediated effects on immunoregulatory mechanisms in DCs. For this purpose, we analyzed expression of programmed death-ligand 1 (PD-L1) and PD-L2 on BM-DCs since both PD-1 ligands play a regulatory role in immune responses to contact allergens. Blocking PD-L1/PD-1 with monoclonal antibodies has been shown to enhance CHS reactions [25,31], while interfering RNA-mediated silencing of PD-L2 on DCs inhibited the elicitation of CHS [32]. Here, we found comparable expression of PD-L1 and PD-L2 on unstimulated and LPS-stimulated *Cnr2^−/−^* and WT BM-DCs (Figure 5a).

Finally, we studied the production of IL-10 by activated *Cnr2^−/−^* and WT BM-DCs. This anti-inflammatory cytokine has previously been shown to efficiently inhibit ear swelling responses in hapten-induced CHS when overexpressed in virally transduced DCs upon in vivo transfer [33]. In our study, we found equivalent levels of IL-10 in the supernatants of stimulated *Cnr2^−/−^* and WT BM-DCs (Figure 5b), underscoring that neither expression of PD-1 ligands nor IL-10 production in BM-DCs in vitro is affected by CB2.

## 3. Discussion

In this study, we investigated the role of CB1 and CB2 in CHS induction by DNFB-haptenized DCs in vivo and the specific influence of CB2 in maturation and migration of DCs in vitro. Our data demonstrate that the absence of CB2 reduced the potential of haptenized DCs to induce CHS responses in mice. Adoptive transfers of DNFB-haptenized *Cnr2*^−/−^ or *Cnr1*^−/−^/*Cnr2*^−/−^ deficient DCs resulted in attenuated ear swelling while transfer of haptenized *Cnr1*^−/−^ DCs did not affect CHS in WT animals. These findings suggest that specifically CB2 affects DCs in a cell-intrinsic manner. Notably, host *Cnr2* deficiency did not alter ear swelling responses, whereas *Cnr1*-deficient recipients showed enhanced allergic responses upon transfer of WT DCs. Thus, CB1 expression by host cells, but not adoptively transferred donor cells, mediates protective effects. These results are in line with our previous findings showing that lack of CB1 expression in host keratinocytes leads to exacerbated CHS responses associated with increased expression of chemokines including CCL8 and the alarmin thymic stromal lymphopoietin TSLP [21,27,28]. With these opposing roles of CB1 (host protection) and CB2 (maturation signal for haptenized DCs) in mind, we investigated changes in key functions of DCs that may be operative in CHS induction by haptenized DCs.

Various chemokines and their receptors have been functionally implicated in migration of DCs into the lymph node during CHS [34,35,36]. Accordingly, it has been demonstrated that CCR7 mediates entry of both dermal and epidermal DCs into the lymphatic vessels and thus acts as a master regulator of DC migration under steady-state and inflammatory conditions [29,37]. Additional studies have highlighted the functional involvement of the CXCL12-CXCR4 axis in CHS [30,38,39]. CHS responses were impaired by CXCR4 antagonist administration during the sensitization phase in mice indicating that CXCL12-CXCR4 engagement was required for migration of cutaneous DCs [30]. Here, we demonstrate that CB2 deficiency did not alter CCR7 and CXCR4 expression levels on unstimulated BM-DCs, but reduced TLR-mediated upregulation of both receptors on activated BM-DCs. This is surprising in the context of an earlier study demonstrating that activation of BM-DCs with a cocktail of TNF, IL-1β, IL-6, PGE2, and the CB2 agonist GP1a did not affect CCR7 upregulation. Here, Adhikary and coauthors harvested BM-DCs on day 7 of culture and further matured them for up to 2 days in the presence of the cytokine cocktail and PGE2 and the CB2 agonist [25]. In our study, however, BM-DCs were utilized at day 6 and stimulated with the TLR agonist LPS according to established maturation protocols. Both maturation protocols induce distinct gene expression profiles and immune related functions in BM-DCs [40]. Thus, the diverse findings from both studies can be explained by differences in the maturation states of BM-DCs.

Furthermore, Adhikary et al. showed that CB2 deficient BM-DCs matured with various cytokines migrated equally well towards CCL19 as WT BM-DCs [25]. In our transwell migration assays, both, unstimulated as well as LPS or CpG-stimulated *Cnr2^−/−^* DCs, showed an equivalent migratory behavior towards CCL19 when compared to WT DCs. However, it is yet unresolved whether CCR7 expression levels potentially altered under in vivo conditions in activated *Cnr2^−/−^* DCs may affect their migratory behavior in CHS.

Additionally, we found that *Cnr2^−/−^* BM-DCs were impaired in TLR ligand-induced upregulation of MHC class II and the co-stimulatory molecules CD40 and CD86. It is possible that these phenotypic changes in *Cnr2^−/−^* DCs affect their capacity to activate T cells in CHS. In this context, blockade of the CD40-CD40L pathway during sensitization has been shown to inhibit T cell mediated responses and thus induce tolerance in murine CHS [41]. CD40-CD40 ligand interactions have further been demonstrated to regulate migration of antigen-bearing DCs from skin to draining lymph nodes in vivo, ameliorating CHS responses in CD40L knockout mice after hapten sensitization [42]. Furthermore, CD86 expression on DCs has been reported to be required for induction of CHS. Accordingly, injection of anti-CD86 antibodies before DNFB sensitization inhibited CHS development associated with reduced upregulation of CD80 and CD86 on DCs in the lymph nodes [43,44]. This involvement of CD86 in CHS was further highlighted by a prior study demonstrating that topical application of cream-emulsified CD86 siRNA in mice after sensitization reduced CD86 expression of DCs in skin-draining lymph nodes and ameliorated the clinical manifestations of CHS [45]. Hence, it is possible that *Cnr2* deficiency in haptenized DCs dampens induction of CHS responses due to reduced upregulation of co-stimulatory molecules, resulting ultimately in impaired induction of effector T cell responses [46].

In conclusion, our findings confirm the importance of CB1 on host cells for protection from CHS and identified CB2 expression on DCs as a factor contributing to the development of the disease. CB2 plays a DC-intrinsic role that affects CHS induction in vivo. Loss of CB2 signaling resulted in reduced upregulation of CCR7 and CXCR4 on activated BM-DCs but did not affect in vitro migration behavior of unstimulated BM-DCs in response to a CCR7 ligand. CB2 deficiency further impaired upregulation of MHC class II and co-stimulatory molecules in DCs under inflammatory conditions in vitro and may thus be operative in DC-dependent mechanisms involved in T cell activation in CHS. Thus, targeting CB2 signaling specifically in DCs has therapeutic potential for the treatment of atopic dermatitis, which represents a considerable burden on patients and healthcare systems.

## 4. Materials and Methods

### 4.1. Animals

Mice with a genetic deletion of the *Cnr1/2* (*Cnr1/2^−/−^*), *Cnr1* (*Cnr1^−/−^*), and *Cnr2* (*Cnr2^−/−^*) gene on the C57BL/6J background [46] and their littermate controls were bred and housed in the Specific Pathogen Free (SPF) animal facility of the House for Experimental Therapy (University of Bonn, Germany) and ZTE Münster. All experiments were conducted according to the institutional and national guidelines for the care and use of laboratory animals and were approved by the local government authorities (Landesamt für Natur, Umwelt und Verbraucherschutz NRW, Germany, date of document: 17/04/2008).

### 4.2. Contact Hypersensitivity

Mature DCs were haptenized with 2,5 mM dinitrobenzene sulfonic acid (DNBS, MP Biomedicals, Solon, OH, USA), the water-soluble analogue of the obligate contact sensitizer 1-fluoro-2,4-dinitrobenzene (DNFB). For sensitization, naïve mice received two inguinal s.c. injections of 5 × 10^5^ haptenized DCs. For elicitation of CHS, ears of mice were painted with 10 µL of 0.3% DNFB on day 5, a second challenge was performed on day 12. Ear thickness was measured 48 h hours after the second challenge using a spring-loaded caliper (Kroeplin, Schlüchtern, Germany). Ear swelling was calculated in each mouse as the difference in ear thickness between the unchallenged and challenged ear.

### 4.3. Generation of BM-DCs

Bone marrow cells were cultured as described previously [47,48]. In brief, bone marrow cells from *Cnr1^−/−^*, *Cnr2*^−/−^ and *Cnr1*^−/−^/*Cnr2*^−/−^ mice were seeded at 5 × 10^5^ cells/mL in DMEM supplemented with 10% FCS, 1% MEM/NEAA, 1% Penicillin/Streptavidin 0.1% β-Mercaptoethanol (all from Thermo Fisher Scientific, Inchinnan, UK) and 10% conditioned medium of B16 cells. Medium was exchanged on day 3 and cells stimulated on day 5 with either 100 ng/mL *Escherichia coli* Lipopolysaccharide (LPS) Serotype 0127:B8 (Sigma-Aldrich, Saint Louis, MO, USA) or 1 nmol/mL CpG1668 (TIB MOLBIOL, Berlin, Germany). After 16 h of stimulation, cells were harvested and analyzed by FACS or transferred in the migration assay.

### 4.4. Transwell Migration Assays

GM-CSF generated BM-DCs were stimulated at day 5 of culturing with LPS (Sigma-Aldrich, Saint Louis, MO, USA) or CpG1668 (TIB MOLBIOL, Berlin, Germany) and 4 × 10^5^ cells transferred at day 6 to the upper chamber compartment of a 5 µm pore size transwell plate (Corning, Kennebunk, ME, USA). The lower well compartments were filled either with media only or 200 ng/mL CCL19 (R&D Systems, Minneapolis, MN, USA). The concentration of 200 ng/mL recombinant CCL19 was selected in accordance with earlier studies demonstrating optimal chemotactic responses at these concentrations [41,48]. Cells were allowed to migrate for 4 h at 37 °C in 5% CO_2_. Cell counts of the migrated DCs harvested from the lower chambers were determined by FACS.

### 4.5. FACS Analysis

Fluorescence staining was performed using the following antibodies: anti-CD11c (AF448 labeled), anti-CD11b (BV510 labeled) anti–I-Ab (BV421 labeled), anti-CCR7 (BV421 labeled), anti-CXCR4 (APC labeled), anti-PD-L1 (APC labeled), anti-PD-L2 (PE labeled) all from Biolegend (San Diego, CA, USA) and anti-CD40 (APC labeled) and anti-CD86 (PE labeled) from eBioscience (San Diego, CA, USA). Fluorescence was analyzed using a FACS Canto™ II (Becton Dickinson, Heidelberg, Germany) flow cytometer and Flowjo™ software (version 10.0.8, FlowJoLLC, Ashland, OR, USA). 

### 4.6. Elisa

IL-10 in cell culture supernatants was measured using ELISAs according to the manufacturer’s instructions (Biolegend, San Diego, CA, USA). Detection limits for IL-10 were 2000 pg/mL.

### 4.7. Statistical Analysis

Statistically significant differences were calculated with the non-parametric Mann-Whitney U-test using SPSS software (version 25, IBM Corp., Armonk, NY, USA) and GraphPad Prism (version 8.00 for Mac OS X, La Jolla, CA, USA). * *p* < 0.05; ** *p* < 0.01.

## Abbreviations

9-THC	9-tetrahydrocannabinol
ACD	Allergic contact dermatitis
AF	Alexa Fluor
APC	allophycocyanin
BM-DCs	Bone marrow derived dendritic cells
BV	Brilliant Violet
CB/Cnr	Cannabinoid receptor
CCL/CXCL	Chemokine ligands of the CC and CXC classes
CCR/CXCR	Chemokine receptors of the CC and CXC classes
CHS	contact hypersensitivity
CpG	CpG Oligonucleotide 1668
DCs	dendritic cells
DMEM	Dulbecco modified Eagle medium
DNFB	2,4-dinitro-1-fluorobenzene
eCBS	Endocannabinoid system
FCS	Fetal calf serum
GM-CSF	granulocyte-macrophage colony-stimulating factor
Ig	Immunoglobulin
IL	interleukin
KO	knock out
LPS	lipopolysaccharide
mAb	monoclonal antibody
2-ME	2-mercaptoethanol
MEM/NEAA	Minimum essential medium/Non-essential amino acids
MHC	Major histocompatibility complex
PE	phycoerythrin
s.c.	subcutaneously
SEM	standard error of the mean
TH	T helper
9-THC	9-tetrahydrocannabinol
TLR	Toll-like receptor
TSLP	thymic stromal lymphopoietin
WT	wild type

## References

[1] Martin S., Esser P., Weber F., Jakob T., Freudenberg M., Schmidt M., Goebeler M. (2011). Mechanisms of chemical-induced innate immunity in allergic contact dermatitis. Allergy.

[2] Peng W., Novak N. (2015). Pathogenesis of atopic dermatitis. Clin. Exp. Allergy.

[3] Carrera Y.I.L., Al Hammadi A., Huang Y.-H., Llamado L.J., Mahgoub E., Tallman A.M. (2019). Epidemiology, Diagnosis, and Treatment of Atopic Dermatitis in the Developing Countries of Asia, Africa, Latin America, and the Middle East: A Review. Dermatol. Ther..

[4] Martin S.F. (2012). Allergic contact dermatitis: Xenoinflammation of the skin. Curr. Opin. Immunol..

[5] Honda T., Egawa G., Grabbe S., Kabashima K. (2013). Update of immune events in the murine contact hypersensitivity model: Toward the understanding of allergic contact dermatitis. J. Investig. Dermatol..

[6] Kissenpfennig A., Malissen B. (2006). Langerhans cells–revisiting the paradigm using genetically engineered mice. Trends Immunol..

[7] Clausen B.E., Stoitzner P. (2015). Functional specialization of skin dendritic cell subsets in regulating T cell responses. Front. Immunol..

[8] Lehtimäki S., Savinko T., Lahl K., Sparwasser T., Wolff H., Lauerma A., Alenius H., Fyhrquist N. (2012). The Temporal and Spatial Dynamics of Foxp3^+^ Treg Cell–Mediated Suppression during Contact Hypersensitivity Responses in a Murine Model. J. Investig. Dermatol..

[9] Kogan N.M., Mechoulam R. (2007). Cannabinoids in health and disease. Dialogues Clin. Neurosci..

[10] Galiègue S., Mary S., Marchand J., Dussossoy D., Carrière D., Carayon P., Bouaboula M., Shire D., LE Fur G., Casellas P. (1995). Expression of central and peripheral cannabinoid receptors in human immune tissues and leukocyte subpopulations. Eur. J. Biochem..

[11] Zimmer A., Zimmer A.M., Hohmann A.G., Herkenham M., Bonner T.I. (1999). Increased mortality, hypoactivity, and hypoalgesia in cannabinoid CB1 receptor knockout mice. Proc. Natl. Acad. Sci. USA.

[12] Matias I., Pochard P., Orlando P., Salzet M., Pestel J., Di Marzo V. (2002). Presence and regulation of the endocannabinoid system in human dendritic cells. Eur. J. Biochem..

[13] Billings S.D. (2019). Common and critical inflammatory dermatoses every pathologist should know. Mod. Pathol..

[14] Park S.-C. (2019). Neurogenesis and antidepressant action. Cell Tissue Res..

[15] Yamamoto W., Mikami T., Iwamura H. (2008). Involvement of central cannabinoid CB2 receptor in reducing mechanical allodynia in a mouse model of neuropathic pain. Eur. J. Pharmacol..

[16] Tschöp J., Kasten K.R., Nogueiras R., Goetzman H.S., Cave C.M., England L.G., Dattilo J., Lentsch A.B., Tschöp M.H., Caldwell C.C. (2009). The cannabinoid receptor 2 is critical for the host response to sepsis. J. Immunol..

[17] Raborn E.S., Cabral G.A. (2010). Cannabinoid inhibition of macrophage migration to the trans-activating (Tat) protein of HIV-1 is linked to the CB2 cannabinoid receptor. J. Pharmacol. Exp. Ther..

[18] Trebicka J., Racz I., Siegmund S.V., Cara E., Granzow M., Schierwagen R., Klein S., Wojtalla A., Hennenberg M., Huss S. (2011). Role of cannabinoid receptors in alcoholic hepatic injury: Steatosis and fibrogenesis are increased in CB2 receptor-deficient mice and decreased in CB1 receptor knockouts. Liver Int..

[19] Alferink J., Specht S., Arends H., Schumak B., Schmidt K., Ruland C., Lundt R., Kemter A., Dlugos A., Kuepper J.M. (2016). Cannabinoid receptor 2 modulates susceptibility to experimental cerebral malaria through a CCL17-dependent mechanism. J. Biol. Chem..

[20] Maccarrone M., Di Rienzo M., Battista N., Gasperi V., Guerrieri P., Rossi A., Finazzi-Agrò A. (2003). The Endocannabinoid System in Human Keratinocytes Evidence That Anandamide Inhibits Epidermal Differentiation through Cb1 Receptor-Dependent Inhibition of Protein Kinase C, Activating Protein-1, and Transglutaminase. J. Biol. Chem..

[21] Karsak M., Gaffal E., Date R., Wang-Eckhardt L., Rehnelt J., Petrosino S., Starowicz K., Steuder R., Schlicker E., Cravatt B. (2007). Attenuation of allergic contact dermatitis through the endocannabinoid system. Science.

[22] Oláh A., Ambrus L., Nicolussi S., Gertsch J., Tubak V., Kemény L., Soeberdt M., Abels C., Bíró T. (2016). Inhibition of fatty acid amide hydrolase exerts cutaneousanti-inflammatory effects both in vitro and in vivo. Exp. Dermatol..

[23] Ueda Y., Miyagawa N., Matsui T., Kaya T., Iwamura H. (2005). Involvement of cannabinoid CB2 receptor-mediated response and efficacy of cannabinoid CB2 receptor inverse agonist, JTE-907, in cutaneous inflammation in mice. Eur. J. Pharmacol..

[24] Koyama S., Purk A., Kaur M., Soini H.A., Novotny M.V., Davis K., Kao C.C., Matsunami H., Mescher A. (2019). Beta-caryophyllene enhances wound healing through multiple routes. PLoS ONE.

[25] Adhikary S., Kocieda V.P., Yen J.-H., Tuma R.F., Ganea D. (2012). Signaling through cannabinoid receptor 2 suppresses murine dendritic cell migration by inhibiting matrix metalloproteinase 9 expression. Blood.

[26] Kemter A.M., Scheu S., Hüser N., Ruland C., Schumak B., Findeiß M., Cheng Z., Assfalg V., Arolt V., Zimmer A. (2015). The cannabinoid receptor 2 is involved in acute rejection of cardiac allografts. Life Sci..

[27] Gaffal E., Cron M., Glodde N., Bald T., Kuner R., Zimmer A., Lutz B., Tüting T. (2013). Cannabinoid 1 receptors in keratinocytes modulate proinflammatory chemokine secretion and attenuate contact allergic inflammation. J. Immunol..

[28] Gaffal E., Glodde N., Jakobs M., Bald T., Tüting T. (2014). Cannabinoid 1 receptors in keratinocytes attenuate fluorescein isothiocyanate-induced mouse atopic-like dermatitis. Exp. Dermatol..

[29] Ohl L., Mohaupt M., Czeloth N., Hintzen G., Kiafard Z., Zwirner J., Blankenstein T., Henning G., Förster R. (2004). CCR7 governs skin dendritic cell migration under inflammatory and steady-state conditions. Immunity.

[30] Kabashima K., Shiraishi N., Sugita K., Mori T., Onoue A., Kobayashi M., Sakabe J.-i., Yoshiki R., Tamamura H., Fujii N. (2007). CXCL12-CXCR4 engagement is required for migration of cutaneous dendritic cells. Am. J. Pathol..

[31] Hitzler M., Majdic O., Heine G., Worm M., Ebert G., Luch A., Peiser M. (2012). Human Langerhans cells control Th cells via programmed death-ligand 1 in response to bacterial stimuli and nickel-induced contact allergy. PLoS ONE.

[32] Furusawa E., Ohno T., Nagai S., Noda T., Komiyama T., Kobayashi K., Hamamoto H., Miyashin M., Yokozeki H., Azuma M. (2019). Silencing of PD-L2/B7-DC by Topical Application of Small Interfering RNA Inhibits Elicitation of Contact Hypersensitivity. J. Investig. Dermatol..

[33] Besche V., Wiechmann N., Castor T., Trojandt S., Hohn Y., Kunkel H., Grez M., Grabbe S., Reske-Kunz A.B., Bros M. (2010). Dendritic cells lentivirally engineered to overexpress interleukin-10 inhibit contact hypersensitivity responses, despite their partial activation induced by transduction-associated physical stress. J. Gene Med..

[34] Castan L., Magnan A., Bouchaud G. (2017). Chemokine receptors in allergic diseases. Allergy.

[35] Moschovakis G.L., Förster R. (2012). Multifaceted activities of CCR7 regulate T-cell homeostasis in health and disease. Eur. J. Immunol..

[36] Sallusto F., Lanzavecchia A. (2000). Understanding dendritic cell and T-lymphocyte traffic through the analysis of chemokine receptor expression. Immunol. Rev..

[37] Averbeck M., Kuhn S., Bühligen J., Götte M., Simon J.C., Polte T. (2017). Syndecan-1 regulates dendritic cell migration in cutaneous hypersensitivity to haptens. Exp. Dermatol..

[38] Stutte S., Quast T., Gerbitzki N., Savinko T., Novak N., Reifenberger J., Homey B., Kolanus W., Alenius H., Förster I. (2010). Requirement of CCL17 for CCR7-and CXCR4-dependent migration of cutaneous dendritic cells. Proc. Natl. Acad. Sci. USA.

[39] Piao W., Xiong Y., Famulski K., Brinkman C.C., Li L., Toney N., Wagner C., Saxena V., Simon T., Bromberg J.S. (2018). Regulation of T cell afferent lymphatic migration by targeting LTβR-mediated non-classical NFκB signaling. Nat. Commun..

[40] Castiello L., Sabatino M., Jin P., Clayberger C., Marincola F.M., Krensky A.M., Stroncek D.F. (2011). Monocyte-derived DC maturation strategies and related pathways: A transcriptional view. Cancer Immunol. Immunother. CII.

[41] Tang A., Judge T.A., Turka L.A. (1997). Blockade of CD40-CD40 ligand pathway induces tolerance in murine contact hypersensitivity. Eur. J. Immunol..

[42] Moodycliffe A.M., Shreedhar V., Ullrich S.E., Walterscheid J., Bucana C., Kripke M.L., Flores-Romo L. (2000). CD40–CD40 ligand interactions in vivo regulate migration of antigen-bearing dendritic cells from the skin to draining lymph nodes. J. Exp. Med..

[43] Nuriya S., Yagita H., Okumura K., Azuma M. (1996). The differential role of CD86 and CD80 co-stimulatory molecules in the induction and the effector phases of contact hypersensitivity. Int. Immunol..

[44] Reiser H., Schneeberger E.E. (1996). Expression and function of B7-1 and B7-2 in hapten-induced contact sensitivity. Eur. J. Immunol..

[45] Ritprajak P., Hashiguchi M., Azuma M. (2008). Topical application of cream-emulsified CD86 siRNA ameliorates allergic skin disease by targeting cutaneous dendritic cells. Mol. Ther..

[46] Buckley N.E., McCoy K.L., Mezey É., Bonner T., Zimmer A., Felder C.C., Glass M., Zimmer A. (2000). Immunomodulation by cannabinoids is absent in mice deficient for the cannabinoid CB2 receptor. Eur. J. Pharmacol..

[47] Ruland C., Renken H., Kuzmanov I., Fattahi Mehr A., Schwarte K., Cerina M., Herrmann A., Otte D.-M., Zimmer A., Schwab N. (2017). Chemokine CCL17 is expressed by dendritic cells in the CNS during experimental autoimmune encephalomyelitis and promotes pathogenesis of disease. Brain Behav. Immun..

[48] Alferink J., Lieberam I., Reindl W., Behrens A., Weiß S., Hüser N., Gerauer K., Ross R., Reske-Kunz A.B., Ahmad-Nejad P. (2003). Compartmentalized production of CCL17 in vivo: Strong inducibility in peripheral dendritic cells contrasts selective absence from the spleen. J. Exp. Med..

